# Cystatin C for gentamicin dosing - a case study

**DOI:** 10.11613/BM.2025.030902

**Published:** 2025-08-15

**Authors:** Tomáš Šálek, Josef Klhůfek, Martin Vodička, Marek Pšenčík

**Affiliations:** 1 Department of Clinical Biochemistry and Pharmacology, Tomas Bata Hospital in Zlín, Zlín, Czech Republic; 2 Institute of Clinical Biochemistry and Diagnostics, Medical Faculty in Hradec Králové, Charles University in Prague, Prague, Czech Republic; 3 Department of Clinical Pharmacy, Tomas Bata Hospital in Zlín, Zlín, Czech Republic

**Keywords:** gentamicins, creatinine, cystatin C, glomerular filtration rate, drug overdose

## Abstract

The study aims to present a case study of a patient with supratherapeutic serum gentamicin concentration. An 83-year-old male was admitted to the Department of Internal Medicine for persistent loss of appetite, decompensated heart failure, and pneumonia. He was treated with 240 mg gentamicin daily alongside ampicillin/sulbactam penicillin antibiotic. The trough gentamicin concentrations and estimated glomerular filtration rate from creatinine (eGFRcrea) and cystatin C (eGFRcys) were performed. The patient had the supratherapeutic trough gentamicin concentration of 2.5 mg/L. eGFRcrea was 62 mL/min/1.73m^2^ and eGFRcys was 25 mL/min/1.73m^2^. The difference between eGFRcrea and eGFRcys was 148%. Falsely high eGFRcrea in elderly patient led to the supratherapeutic gentamicin concentration even after the standard 240 mg gentamicin dose.

## Graphical abstract

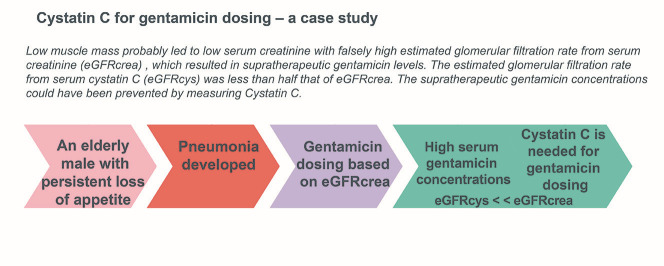

## Highlights

• Serum creatinine concentration strongly depends on muscle mass

• Low muscle mass leads to glomerular filtration rate (GFR) overestimation from serum creatinine

• Cystatin C does not depend on muscle mass

• Cystatin C is needed for dosing of drugs that are excreted by GFR

## Introduction

The glomerular filtration rate (GFR) is a key factor for dosing drugs excreted by the kidney. The kidneys excrete aminoglycoside antibiotics, which are nephrotoxic and can lead to kidney failure in overdosed patients. Once-daily dosing of gentamicin is also recommended based on GFR and body weight (*1*).

Traditionally, plasma creatinine has been used to estimate GFR. Unfortunately, low muscle mass, which is common in elderly, chronically ill patients, affects plasma creatinine. Cystatin C is another marker of GFR, that is not affected by low muscle mass and predicts mortality (*2*).

Patient safety is the reason we are presenting a case study with a significant discrepancy between the estimated glomerular filtration rate from serum creatinine (eGFRcrea) and cystatin C (eGFRcys) that would impact gentamicin dosing.

## Case report

An 83-year-old male was admitted to the Department of Internal Medicine for decompensated heart failure, persistent loss of appetite, and pneumonia. His body weight was 70 kg, height was 170 cm, and his body mass index was 22 kg/m^2^. He lost 5 kg last month and underwent a nutritional specialist consultation. He suffered from chronic heart failure, paroxysmal atrial fibrillation, hypercholesterolemia, hypertension, and chronic kidney disease stage G3a based on eGFRcrea. His regular daily medication included atorvastatin 80 mg, ezetimibe 10 mg, ramipril 1.25 mg, nebivolol 5 mg-0-2.5 mg, rivaroxaban 20 mg, empagliflozin 10 mg, 25-hydroxyvitamin D3, and spironolactone 12.5 mg.

During this hospitalization, he was administered 240 mg of gentamicin daily along with ampicillin/sulbactam penicillin antibiotic. The target trough concentration is below 1 mg/L to reduce nephrotoxicity (*1*). Almost all patients taking gentamicin are monitored for trough concentrations. Clinical pharmacologists (who graduated from a Medical faculty and specialized in Clinical pharmacology) or clinical pharmacists (who graduated from a Faculty of pharmacy and specialized in Clinical pharmacy) manually enter interpretative comments recommending the next gentamicin dosing into the laboratory information system. Fasting blood samples were collected in 5 mL VACUETTE tubes with red top and separator aktivator for clotting (Greiner Bio-One Gmbh, Kremsmünster, Austria), at 6 a.m. Centrifugation at 1500xg for 10 minutes was performed within one hour after sample collection.

Both creatinine and cystatin C were measured using standardized methods traceable to international standards NIST SRM 967 and DA ERM 471, respectively (*3*, *4*). Chronic Kidney Disease Epidemiology Collaboration (CKD-EPI) equations were used to calculate eGFRcrea, eGFRcys, and eGFR from both markers (eGFRcrea+cys) (*5*, *6*). These equations are among the validated formulas according to KDIGO 2024 guidelines (*7*). For our patient, the following equations were used: CKD-EPI creatinine 2009 equation: eGFRcrea = 141 x (creatinine/79.6)^-1.209^ x 0.993^Age^, CKD-EPI cystatin C 2012 equation: eGFRcys = 133 x (cystatin C/0,8)^-1.328^ x 0.996^Age^, CKD-EPI creatinine-cystatin C 2012 combined equation: eGFRcrea+cys = 135 x (creatinine/79.6)^-0.601^ x (cystatin C/0.8)^-0.711^ x 0.995^Age^.

All laboratory tests, including thyroid-stimulating hormone (TSH) and albumin, were measured on an Abbott Architect ci 16200 automated analyzer (Abbott Laboratories, Abbott Park, USA). Albumin was measured using a bromocresol green photometric method. TSH was measured using a chemiluminescent microparticle immunoassay calibrated against WHO TSH 80/558 reference material.

All dosing data were used to interpret serum gentamicin concentrations, including the initial dose date, dosing time, 240 mg gentamicin dose, dosing interval, and intravenous route of administration (*8*). Before the fourth dose, his trough concentration had increased to 2.5 mg/L. Gentamicin concentrations before the second and third doses were not ordered. The modeling program MwPharm++ 1.7.14.0 version (Mediware, Groningen, The Nederlands) also supported dosing using a population pharmacokinetic two-compartment model for gentamicin. Based on this model, gentamicin concentrations were best fitted when eGFRcys was used to estimate renal function. The interpretative comment on the laboratory report recommended extending the dosing interval to 36 hours and reducing the dose to 160 mg/L, which resulted in a trough concentration of 1.0 mg/L. Laboratory test results are shown in Table 1.

**Table 1:** Laboratory fasting serum test results in the patient with supratherapeutic gentamicin concentrations, monitoring of inflammation and renal function

**Serum laboratory test (unit)**	**Result** **August 14th**	**Result** **August 18th**	**Result** **August 28th**	**Reference range**
Sodium (mmol/L)	137	138	142	136-144
Potassium (mmol/L)	4.7	4.7	4.1	3.8-5.1
Chloride (mmol/L)	104	107	104	95-107
Glucose (mmol/L)	4.9	4.8	4.9	3.9-5.5
Gentamicin (mg/L)	2.5	1.0	-	< 1.00
Serum urea (mmol/L)	9.0	9.0	14.4	3.0-8.0
Creatinine (µmol/L)	96	112	138	49-90
eGFRcrea (mL/min/1.73m^2^)	62	52	40	90-150
Cystatin C (mg/L)	2.21	2.41	-	< 0.96
eGFRcys (mL/min/1.73m^2^)	25	22	-	90-150
eGFRcrea+cys (mL/min/1.73m^2^)	38	33	-	90-150
Albumin (g/L)	26.5	25.1	26.6	36.0-45.0
C-reactive protein (mg/L)	136	91	23	0-2
Thyroid stimulating hormone (mU/L)	0.725	-	-	0.350-4.940
eGFRcrea - estimated glomerular filtration rate from serum creatinine. eGFRcys - estimated glomerular filtration rate from serum cystatin C. eGFRcrea+cys - estimated glomerular filtration rate from serum creatinine and cystatin C.				

After successful antibiotic treatment, the patient was discharged home on August 28th.

The patient signed the informed consent for the publication of his case report. The local Ethics Committee approved the publication of case study No.2024/18.

## Discussion

The patient with supratherapeutic gentamicin concentration and a significant difference between eGFRcrea and eGFRcys was presented. Gentamicin concentrations were best fitted when eGFRcys was used to estimate renal function.

Groothof *et al.* in their large study involving over 13,000 patients, reported that serum creatinine is an unreliable marker of GFR due to its dependence on muscle mass (*9*). Our elderly patient likely had a falsely overestimated eGFRcrea due to low muscle mass.

Hanna *et al*. analyzed a cohort of 1869 adults with cancer. Their mean age was 66 years. A total of 543 patients who had more than 30% lower eGFRcys compared with eGFRcrea had more commonly trough supratherapeutic concentrations of vancomycin and digoxin, hyperkalemia related to trimethoprim-sulfamethoxazol, and toxic effects of baclofen (*10*). Our patient also had more than 30% lower eGFRcys and supratherapeutic trough concentrations of drug excreted by kidneys.

Rebollo and Cepeda-Piorno presented a case study of a 78-year-old male patient with supratherapeutic trough amikacin concentrations which can lead to nephrotoxicity and ototoxicity. His body mass index was 29 kg/m^2^. They concluded that eGFRcys, based on the CKD-EPI 2012 equation, is better for dosing amikacin and aminoglycosides than eGFRcrea (*11*). This is consistent with our findings of supratherapeutic gentamicin trough concentrations, and gentamicin concentrations were best fitted when eGFRcys was used to estimate renal function.

Chin *et al.* found that eGFRcrea+cys calculated using the CKD-EPI 2012 equation provided the best estimate of gentamicin clearance. Their study group included 260 patients with a median age of 67 years (*12*). The disadvantage of this study is that creatinine was not measured using a standardized enzyme method traceable to international standards NIST SRM 967. Creatinine was measured by a modified Jaffe reaction.

Karimzadeh *et al.* conducted a systematic review on toxicity in patients receiving repeated doses of aminoglycosides and once-daily dosing. Once-daily dosing was found to be safer in terms of nephrotoxicity (*13*), which is why we also used safer once-daily dosing.

The KDIGO 2024 guidelines recommended the eGFRcrea+cys equation to confirm for chronic kidney disease, determine the stage, and adjust medication dosing for renally excreted drugs with a narrow therapeutic index (*7*). The reason for using the combined equation is that cystatin C may be affected by variables such as thyroid disease, corticosteroid use, and cancer. We did not have these non-renal variables in our patient.

Chen *et al.* analyzed the UK Biobank and found that older age and male gender were strong predictors for the group with lower eGFRcys compared to the group with eGFRcrea. The group with lower eGFRcys had a 3-fold higher prevalence of diabetes and current smoking and a 2-fold higher prevalence of cardiovascular disease compared to the group with lower eGFRcrea (*14*). This is consistent with the case of our patient, who had cardiovascular disease including heart failure.

Wang *et al.* reported that cystatin C was positively associated with cardiovascular and all-cause mortality (*15*). This finding also highlights the role of cystatin C in clinical diagnostics for prognostic purposes.

Šálek *et al.* reported that measuring cystatin C would avoid supratherapeutic concentrations of gentamicin and digoxin. The median difference between eGFRcrea and eGFRcys in patients in the Intensive Care Unit was 19 mL/min/1.73m^2^ (46%) (*16*, *17*). Our patient also had trough supratherapeutic gentamicin concentrations and a significant difference between eGFRcrea and eGFRcys. Muscle wasting in critically ill patients is common. Our patient had pneumonia, which is also an acute and potentially fatal diagnosis. These manuscripts emphasize that this situation is quite common among elderly patients.

Hu *et al.* demonstrated that cystatin C was positively associated with inflammatory markers CRP and procalcitonin in patients with sepsis (*18*). Our patient also had high CRP concentrations. On the other hand, his gentamicin concentrations were best fitted when eGFRcys was used to estimate renal function.

A limitation of this case study is that we did not measure GFR using the gold-standard exogenous marker method like inulin clearance. It could show true biases between inulin clearance and eGFRcrea, eGFRcys, and eGFRcrea+cys. On the other hand, cystatin C is a newly emerging global standard for eGFR (*19*).

**Figure 1:**
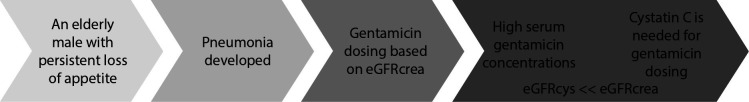
The overall incorrect dosing process of gentamicin. Low muscle mass probably led to low serum creatinine with falsely high estimated glomerular filtration rate (eGFRcrea) from serum creatinine, which resulted in supratherapeutic gentamicin concentrations. The estimated glomerular filtration rate from serum cystatin C (eGFRcys) was less than half that of eGFRcrea. The supratherapeutic gentamicin concentrations could have been prevented by measuring cystatin C.

The results of this case study can be interpreted as follows: low muscle mass likely led to low serum creatinine with falsely high eGFRcrea, resulting in supratherapeutic gentamicin concentrations. This situation could have been prevented by measuring cystatin C. Figure 1 shows the total incorrect dosing process.

We can conclude that eGFRcys plays a key role in the care of patients taking drugs eliminated by the kidneys. Estimating GFR without cystatin C would lead to supratherapeutic concentrations and could affect patient safety.

## Data Availability

All data generated and analyzed in the presented study are included in this published article.
